# The predictive power of employment trajectories on cognition of older adults: Evidence from Chile

**DOI:** 10.1016/j.socscimed.2025.118281

**Published:** 2025-05-29

**Authors:** Magdalena Delaporte

**Affiliations:** a Population Studies Center and Department of Sociology, University of Pennsylvania, 3718 Locust Walk, Philadelphia, PA, 19104, United States; b Population Aging Research Center, University of Pennsylvania, United States; c International Max Planck Research School for Population, Health and Data Science, Max Planck Institute for Demographic Research, Germany; d Leonard Davis Institute of Health Economics, University of Pennsylvania, United States

**Keywords:** Aging, Cognition, Employment, Life-course, Chile

## Abstract

Chile’s population is rapidly aging, with a notable increase in the older population over recent decades. The growth in the proportion of older individuals has substantial implications for physical and cognitive health, healthcare expenditures and policies, given the escalating burden of age-related health conditions. Therefore, it is critical to have a deeper understanding of factors that predict healthy aging. This study explores the relationships between employment trajectories and later-life cognitive function among Chilean adults. Using data from a sample of Chilean adults aged 60–79, this study examines employment histories spanning 36 years (1980–2015) and their associations with cognitive outcomes assessed in 2019. Applying Group-Based Trajectory Modeling (GBTM), I identify distinct employment trajectories for women and men, which I then link to cognitive function through linear regressions. Findings suggest that individuals with more sustained labor-force participation exhibit better cognitive outcomes, particularly in memory and executive function domains, with notable differences by gender. Specifically, women entering the labor-force later in life display better cognitive performance compared to women with low participation in the labor-force throughout adulthood, while men with extended employment histories show positive associations with cognition regardless of whether they exit the labor-force around retirement age or not. These findings highlight the potential cognitive advantages of prolonged employment, contributing to research on social determinants of cognition in later life. This study offers a critical input for labor, health and old-age pension policies, in an aging population such as in Chile.

## 1. Introduction

Research has long shown that employment is associated with health (Braveman et al., 2011; Elo, 2009). In particular, participating in the labor-force has been found to be positively linked to several health outcomes, such as physical functioning and self-rated health (Devillanova et al., 2019; P. McDonough et al., 2017; Ross and Mirowsky, 1995; Zella and Harper, 2021). These associations can be understood through the social-determinants-of-health framework, which emphasizes the importance of stable employment for financial security, access to healthcare, and social engagement in influencing health (Braveman et al., 2011). Even though much of the literature has focused on physical health, there is evidence that employment stability is also linked with better mental health outcomes (Alegría et al., 2018; Allen et al., 2014; Friedland and Price, 2003; P. Virtanen et al., 2005).

Fewer studies have focused on the role of employment in shaping cognitive function in later life. Cognition can be disaggregated into two cognitive processes: fluid and crystallized cognition. Fluid cognition is associated with abstract and logical thinking and declines with age (Shakeel and Goghari, 2017). Crystallized intelligence, related to cultural knowledge and experience, tends to remain stable during adulthood (Kaufman and Horn, 1996). Declines in both types of cognition have been associated with Alzheimer’s Disease and Related Dementias (AD/ADRD) (Matsuda and Saito, 1998; I. M. McDonough et al., 2016), one of the leading causes of disability and dependence at older ages (Alzheimer’s Disease International, 2014). With the number of people suffering AD/ADRD projected to increase rapidly globally (Sosa-Ortiz et al., 2012), cognitive decline will pose significant challenges to healthcare and pension systems. Understanding how life-course processes, such as employment histories, predict cognition among older adults can provide insights regarding factors that play a role in older adults’ cognitive health.

Research from high-income countries suggests that employment and cognitive demands at work are important predictors of cognitive skills and cognitive decline (Andel et al., 2005; Kajitani et al., 2017; Kobayashi and Feldman, 2019; Pool et al., 2016; Wahrendorf et al., 2019, 2021). In particular, literature indicates that employment is relevant for fluid cognition, while it might be less important for crystallized cognition (M. Virtanen et al., 2009). Moreover, the association between labor-force participation and cognition appears to differ by gender. For example, women in European countries benefit from part-time employment, contrary to men (Bertogg and Leist, 2023; Ice et al., 2020). In addition, regardless of marital and parental status, women experience slower memory declines with extended periods of labor-force engagement (Mayeda et al., 2020). Evidence regarding the role of employment in cognitive health at older ages is also emerging for low- and middle-income countries. Yu et al. (2024), for instance, find that midlife employment is associated with higher memory scores in South Africa, suggesting that the role of employment in cognitive aging may extend beyond high-income settings.

Despite such emergent evidence, gaps remain in our understanding of the association between long-term employment and cognition at older ages. First, although some studies have incorporated long-term employment trajectories, many still use either a single measure of labor-force participation or short employment histories (Andel et al., 2005; Gutierrez et al., 2024; Pool et al., 2016; Rusmaully et al., 2017; Yu et al., 2024). Second, most literature has focused predominantly on the United States and Europe, with limited evidence for other contexts. To address these gaps, this paper explores the associations between employment histories over 36 years and later-life cognitive function for older Chileans, using a comprehensive assessment of multiple cognitive domains, and for which the role of employment trajectories has not been previously studied.

As the global population ages globally, it is important to study aging and cognition across diverse economic and demographic contexts. Chile, in particular, is an interesting case. As it transitioned into a high-income country, it began facing important pressures associated with an aging population, such as a rise in the old-age dependency ratio, which stood at 18.4 % in 2021, exceeding substantially the South American average of 13.9 % (United Nations, 2022). Alongside these demographic shifts, Chile also underwent economic changes that affected the life-course trajectories of today’s older adults. In particular, they experienced their early childhood and adolescence in the context of a middle-income country; life stages that are crucial for shaping educational and employment opportunities (Currie and Vogl, 2013) and cognitive outcomes in later life (Cheng et al., 2025). During their working years, however, these individuals benefited from new employment opportunities and health-care access brought by the expansion of the Chilean economy (Núñez et al., 2020; Parro and Reyes, 2019), which might have provided protection for cognitive functions (Frenz et al., 2014). Nevertheless, the nature of these employment opportunities might have been affected by other labor market conditions in Chile, such as high levels of informal employment (OECD, 2023) and gender disparities in labor-force participation (Contreras et al., 2011), both of which may influence cognitive aging.

Chile provides a unique setting to examine the role of employment trajectories on cognitive outcomes at older ages. Unlike experiences from other high-income countries, the recent Chilean economic and demographic transitions might reflect a broader global trend characterized by the simultaneous experience of population aging and economic development. Understanding how long-term employment patterns are associated with cognitive function in older adults can provide insights into the broader health implications of labor-market experiences in Chile and other developing economies.

## 2. Methods

### 2.1. Data

The data used in this study come from two sources: the Chilean Social Protection Survey (SPS) and the Chile-Cog study. The SPS is a longitudinal study representative of Chileans aged 18 and above that started in 2002. Initially focused on affiliates of the pension system, its sample was expanded in 2004 to make it representative of the entire adult population. Follow-up waves were conducted in 2004, 2006, 2009, 2016 and 2019/2020, with a refresher sample included in 2016 to offset attrition. SPS collects data on income, employment and health, among others socioeconomic indicators. In 2017, a supplementary survey called ENCAVIDAM was administered to SPS respondents aged 60 and older to assess their health and quality of life.

The second data source is the Chile-Cog study, which was conducted in 2019 to assess cognition among Chilean older adults. In particular, the Chile-Cog sample was drawn from ENCAVIDAM participants and supplemented with SPS respondents who turned 60 between 2017 and 2019. As a result, the Chile-Cog sample (N = 2033) is representative of Chileans aged 60 years and older. Chile-Cog was carried out between September and December in 2019 and consisted of an in-person assessment conducted by trained interviewers. More information on the Chile-Cog data can be found in this link: https://web.sas.upenn.edu/harmonized-cognitive-assessment-protocol-chile. SPS datasets can be merged to Chile-Cog, allowing the linkage of employment trajectories up until 2016 with measures of cognitive function in 2019.

#### 2.1.1. Analytical sample

For this analysis, I restrict the sample to Chile-Cog respondents who participated in the SPS 2016 wave (N = 1992). This ensures that all respondents have employment information up to 2016, the most recent SPS wave before Chile-Cog data collection. Including respondents who did not participate in 2016 would have generated missing employment data between their last SPS interview and 2016. Most of Chile-Cog respondents interviewed in 2016 participated in at least three of the four SPS waves between 2002 and 2009 (94.8 %).

I further restrict the sample to Chile-Cog respondents aged 60–79 years old in 2019 (N = 1629). Given that I estimate employment trajectories since 1980 (which is described with more detail in the following subsection), it is possible that older adults in the Chile-Cog sample were not part of the labor-force during the initial years covered by the data. For example, a 90-year-old woman in 2019, who was age 51 in 1980 and whose employment trajectory may have ended with her retirement in 1991, could have a significantly different trajectory from a 65-year-old woman in 2019 who was 26 years old in 1980. By narrowing the sample to respondents aged 60 to 79 in 2019, I focus on individuals who were between the ages 21 and 40 in 1980, ensuring more comparable employment trajectories for predicting their cognitive outcomes in later life.

Finally, I exclude respondents with missing data in cognitive scores or covariates. Missing cognitive scores are primarily due to illiteracy (N = 109) or very low cognitive scores that prevented the respondent from completing the interview (N = 9). The final analytical sample consists of 845 women and 663 men. A flowchart with the sample selection criteria and final number of observations by sex is in Fig. A1 in Appendix.

### 2.2. Measures

#### 2.2.1. Cognition

Chile-Cog applied, in 2019, the Harmonized Cognitive Assessment Protocol (HCAP). This instrument was originally designed by the Health and Retirement Study (HRS) team (Institute for Social Research, 2023) and later adapted to the Mexican context as Mex-Cog (Mejia-Arango et al., 2020). Chile-Cog was designed to be comparable to the Mex-Cog instrument with some adaptations for differences between the Mexican and Chilean contexts. The instrument includes a battery of questions intended to evaluate the cognitive status of the respondents in five domains: (1) *Orientation* assesses the respondent’s awareness of time and place. Chile-Cog questions from this domain include identifying the current date and location of the interview. (2) *Memory* is the ability to remember words and stories, including recalls of 3 and 10 words and a short and long story, and word recognition. (3) *Executive function* covers higher-order cognitive processes that involve planning, problem-solving, mental flexibility, and multi-tasking. Items in this domain include grouping similar words and identifying symbols and digits, among others. (4) *Language* evaluates language abilities, including naming, reading and writing. (5) *Visuospatial* measures the ability to coordinate motor function with visual perception. Chile-Cog participants were asked to draw figures (circles, pentagons, and a cube, among others).

For this analysis, I use two dependent variables to measure cognition. First, I use a total cognitive score that considers items across the five cognitive domains that are assessed in Chile-Cog. Additionally, I use the 30-point version of the Mini-Mental State Examination (MMSE), which has been validated and previously used in Chile as a summary measure of cognitive function (Quiroga et al., 2004).

In addition to the total score, I examine associations between employment trajectories and the five specific cognitive domains to explore whether these relationships vary by domain. Although these domains capture different aspects of cognitive function that relate to both fluid (i.e., immediate and delayed recall memory) and crystallized cognition (i.e., language abilities), it is not possible to empirically isolate these two processes with the available data.

For all the estimations, I standardize scores to have a mean of 0 and a standard deviation of 1. Moreover, I use a version of the Chile-Cog data prepared by the Gateway to Global Aging (2023) that imputes missing values for reasons such as refusals, physical impairments, and visual impairments. However, illiteracy is not imputed, so the total score still has missingness for individuals who cannot read or write.

#### 2.2.2. Employment

In each SPS wave, respondents were asked to complete their employment history since their last interview. In 2002, and for new respondents in each wave, employment histories since 1980 were asked. If a respondent did not participate in a specific wave, they were asked to report their employment histories between the last wave they participated in and the current interview. For this study, I use all the waves between 2002 and 2016; therefore, I have employment information between 1980 and 2016. Besides labor-force participation, SPS provides information on job informality and the number of hours worked, among other variables.

To establish an annual indicator of employment status, I utilize employment histories through the end of December 2015. This approach is necessary because the SPS 2016 survey was conducted between April and August of 2016, making it impossible to ascertain the employment status of respondents for the entire year 2016. Labor-force participation is coded as 1 if the respondent worked for pay at least six months in a given year. Thus, I have information on labor-force participation for 36 years (between 1980 and 2015).

In supplementary analysis, I estimate trajectories for informal and for full-time employment. Job informality is defined as not contributing to pension accounts, as used in the literature (Madero-Cabib and Biehl, 2021). As with labor-force participation, job informality is coded as 1 when the respondent worked informally for six months or more each year. Full-time employment is coded as 1 for a specific year if the respondent holds a job requiring at least 40 h per week for a minimum of six out of the 12 months in that year. These analyses provide additional information on pathways through which employment and cognition are associated.

#### 2.2.3. Covariates

I include other covariates to control for characteristics that have been shown to predict cognitive function in previous literature, such as age in 2019 and age-squared, and grades of educational attainment reported in 2016 (Arce Rentería et al., 2019; Rusmaully et al., 2017; Valenzuela and Sachdev, 2006). Grades of schooling attainment is defined to be the highest completed grade (0–12) for individuals who completed secondary school or less. For individuals who attended higher education, it captures the highest completed level (for example, 17 for someone who completed a 5-year college degree). This measure does not account for time spent in school, as respondents may have repeated grades or changed degrees, but rather the highest level completed. I also include grades of educational attainment squared, because I hypothesize the association between education and cognition to be nonlinear. Specifically, the benefit of completing an additional grade is likely higher at lower levels of education (for example, moving from first to second grade) than at higher levels (moving from 11th to 12th grade) (Barnes et al., 2011; Liew and Ang, 2024). Moreover, research has found that early life conditions are linked to later-life cognition (Everson-Rose et al., 2003). Given that parental education has a high proportion of missingness in the sample (16.7 %), I do not use parental education to represent early life conditions. Instead, I use self-reported childhood economic situation to account for economic conditions during early life. SPS respondents were asked: “*How would you describe the economic situation of the household where you grew up?”*, with four response options: *very poor*, *poor*, *good* and *very good*. Individuals who report poor or very poor are classified as having experienced a poor childhood. Childhood socioeconomic status (SES) is reported by new respondents in each wave of the SPS; thus, the data originates from the 2002, 2004 or 2016 waves, depending on when each respondent entered the sample.

In addition, I control for lifelong marital status in 2016 as it has been linked to cognitive function (Liu et al., 2020) and is related to employment trajectories, especially for women (van der Klaauw, 1996). I operationalize it as a binary variable equal to 1 if the respondent has never been married. Lastly, I control for physical limitations measured by a binary variable equal to 1 if the respondent reports at least one limitation in Activities of a Living (ADLs) in the 2016 SPS wave. Including this variable provides an overall measure of physical health, as it has been shown to associate with cognition (Rajan et al., 2013).

### 2.3. Statistical analysis

#### 2.3.1. Descriptive analysis

To characterize the sample, I report the mean values for the relevant variables separately for women and men.

#### 2.3.2. First stage: identifying employment trajectories

I employ the Group-Based Trajectory Model (GBTM) methodology developed by Nagin (2009) to model and predict trajectories of labor-force participation over time. Previous research has used GBTM to understand different aspects of socioeconomic dynamics and their associations with health outcomes (Frech and Damaske, 2019; Nyberg et al., 2019; Yang and Kim, 2023).

GBTM operates by identifying latent trajectories with observed data. Let *Y*_*i*_ = {*y*_*i*1_, *y*_*i*2_, …, *y*_*iT*_} be employment status for individual *i* and *age* = {*age*_*i*1_, *age*_*i*2_, …, *age*_*iT*_} be individuals’ ages in the periods *t* = {1,2, …, *T*}. The likelihood of observing employment status *Y*_*i*_ is given by:

(1) $$P\left(Y_{i}|a_{i}\right)=\sum_{j}^{J}\pi_{j}\cdot P\left(Y_{i}|{\text{age}}_{i},j\right)$$

where *π*_*j*_ is the probability of belonging to group *j* and *P* (*Y*_*i*_ | *age*_*i*_, *j*) is the probability of having outcome *Y*_*i*_ given membership in group *j*. The model assumes that *y*_*it*_ is independent, conditional on being part of group *j* over time *t*. Thus, the likelihood of observing employment status *Y*_*i*_ conditional on membership in group *j* is:

(2) $$P\left(Y_{i}|a_{i},j\right)=\prod^{T}p\left(y_{\text{it}}|{\text{age}}_{\text{it}},j\right)$$

GBTM models *y*_*it*_ as a polynomial function of age. In this paper, I use logit distributions to estimate employment status, since it is coded as a binary variable equal to 1 if respondent *i* works at age *t*. Labor-force participation is modeled as follows:

(3) $$y_{\text{it}}=\beta_{0}^{j}+\sum_{p=1}^{P}\beta_{p}^{j}\cdot {\text{age}}_{\text{it}}^{p}+\varepsilon_{\text{it}}$$

where $\beta_{0}^{j}$ and $\beta_{p}^{j}$ determine the location and shape of each trajectory, given polynomial order *p*.

To obtain the group-based trajectories, I use the Stata plugin *traj* (Jones and Nagin, 2013), which estimates for all respondents the probability of membership in each group. To classify group membership, I assign individuals to the group *j* for which they have the highest probability of membership.

For both women and men, I consider two, three, four or five groups, using either a quadratic or cubic polynomial for age. I conduct a model comparison using the Bayesian Information Criterion (BIC) as recommended by Nagin (2009) to determine the optimal number of groups and polynomial order for age. To improve statistical power, I restrict samples for each group to be at least 5 % of the total sample.

In supplementary analysis, I use GBTM to identify trajectories for informal employment and for full-time employment, both for women and men separately. As with labor-force participation, I use logit models and consider up to five trajectories with either a quadratic or cubic polynomial for age.

#### 2.3.3. Second stage: estimating cognitive function

Following the trajectory estimation, I employ a linear regression model to explore the relationship between cognitive status and membership in each trajectory group. The regression model is specified as:

(4) $$C_{i}=\alpha +\sum_{j=1}^{J}\beta_{j}\cdot G_{\text{ji}}+\gamma \cdot Z_{i}+\varepsilon_{\text{it}}$$

Here, *C*_*i*_ represents the standardized cognitive score obtained from the cognitive assessment battery, encompassing the Mini-Mental State Examination (MMSE), the total cognitive score or the domain-specific score. The trajectory groups *G*_*ji*_, that come from the first stage, are incorporated into the model. In this case, *G*_*ji*_ is equal to one if individual *i* has the highest probability of being in group *j*. *Z*_*i*_ represents the individual characteristics age, age squared, grades of educational attainment and grades of educational attainment squared, childhood socioeconomic status, never married and functional limitations. It is crucial to note that group membership so defined is mutually exclusive; an individual cannot belong to multiple groups simultaneously.

In supplementary analysis, I use the informal and part-time employment groups as independent variables in order to assess their relationships with cognitive function.

## 3. Results

### 3.1. Descriptive statistics

Table 1 shows descriptive statistics for the 845 women and 663 men respondents for whom data are complete. The average woman in the sample is 67.7 years old and has 8.3 completed grades of schooling, while for men the averages are 67.4 years of age and 8.8 completed grades of schooling. On average individuals have almost completed the nine grades required to complete middle school (in some contexts called junior high school or lower-secondary school). In addition, 46 % of women respondents report having poor or very poor socioeconomic status in their childhood, compared to 52 % of men respondents. Moreover, around 25 % of women report, in 2016, having difficulty with at least one Activity of Daily Living (ADL), such as getting dressed or climbing stairs, which is higher than for men (14 %).

Table 1 reports that the average number of years that the women in this sample have been employed through 2015 is 10.8. These women spent on average, 7.3 years in informal employment and 12.0 years in full-time jobs. Men in the sample spent 29.8 years on average in the labor-force between 1980 and 2015. They had informal employment for 12.5 years on average, and they worked full-time jobs for 25.9 years. As expected, men in this sample had more-active participation in the labor-force compared to women, who worked a smaller number of years on average and spent less time in full-time employment.

Lastly, as shown in Table 1, women and men perform similarly on average in the Mini-Mental State Examination (MMSE) with average scores of 25.5 and 25.8 respectively. There are, however, some differences in the overall cognitive scores, where women perform slightly better on average than men. For the specific domains, women have higher scores on average in memory, but lower scores on average in executive function, compared to men. There are no differences between women and men in average scores for orientation, language and visuospatial.

More information on standard deviations and ranges for socio-demographic and health characteristics, as well as employment and cognition can be found in Table A1 and A2 in the Appendix, separately for women and men.

### 3.2. First stage: employment trajectories profiles

**Fig. 1.**
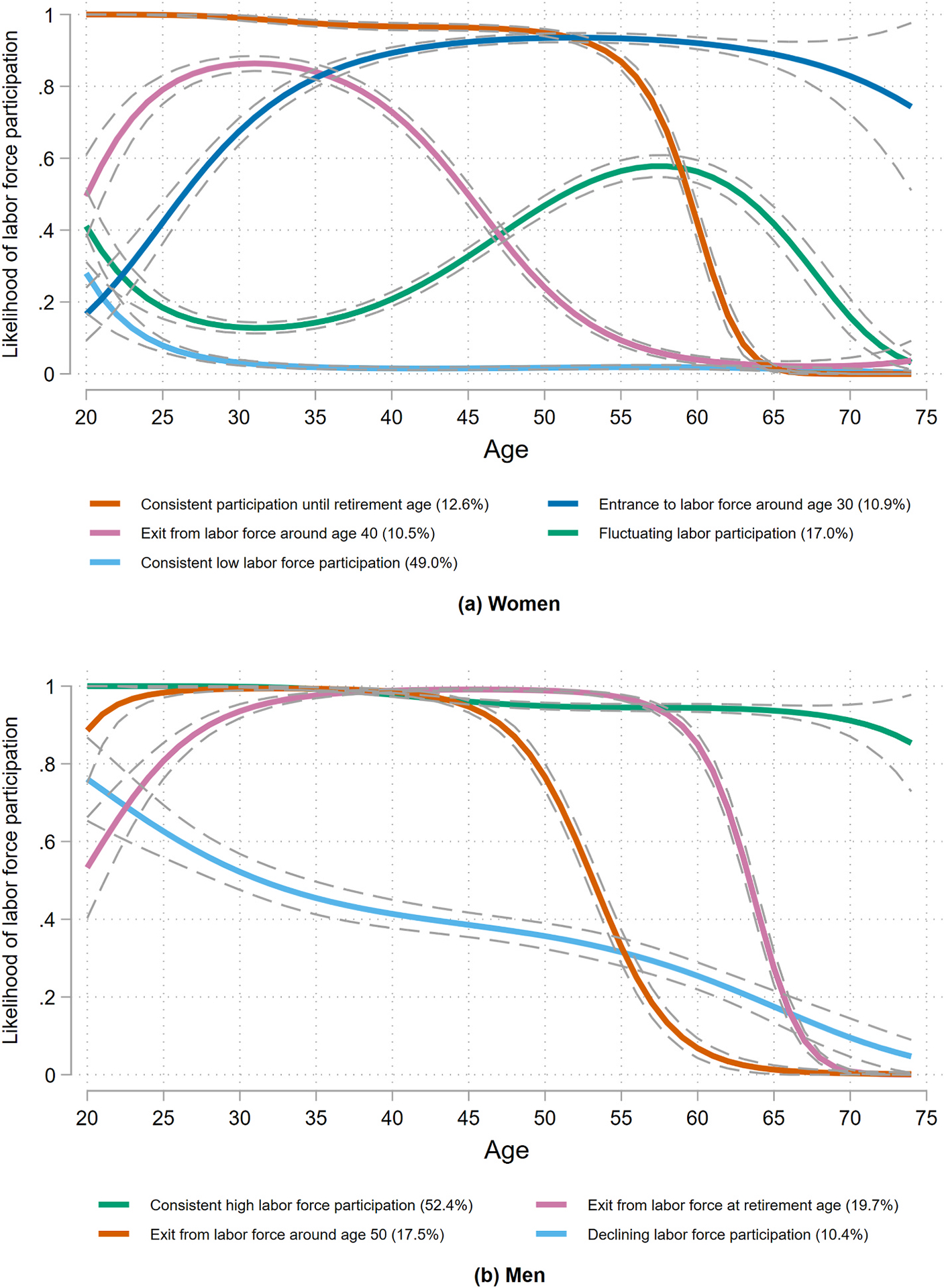
Labor-force participation trajectories Notes: (i) Distributions are weighted using survey weights.

As suggested in Table 1, women and men engage differently with the labor market. Because of this difference, I estimate employment histories separately by sex. GBTM estimation results in identifying five distinct labor-force participation trajectories with a cubic polynomial for age for women and four trajectories with a cubic term for men. BIC values are in Table A3 in Appendix Table A4 in the Appendix includes the average posterior probabilities for the selected models, as well as the entropy values. These values suggest that the model fit is good. The trajectories and their confidence intervals are shown in Fig. 1.

For women, the preferred specification consists of five groups, each with a cubic relationship with age. The first trajectory includes 12.6 % of the women in the sample and represents consistent participation in the labor-force until retirement age (60 years old for women). This group has a high likelihood of being employed during early adulthood, but this likelihood falls after age 55, when they get closer to retirement age. The next group (10.9 %) represents women for whom the probabilities of being in the labor market increase at around age 30 and who continued to work thereafter. I hypothesize that these women entered the workforce after childbearing. Next, we see the trajectory characterized by exit from the labor market at around age 40, making up 10.5 % of the women, who left paid work before the minimum legal retirement age (60 years old). Another 17.0 % of the women adhere to a “fluctuating” trajectory, in which the likelihood of being employed is low until around age 45 and increases in midlife. Lastly, 49.0 % of women follow a “low-participation” trajectory, characterized by a low likelihood of being part of the labor-force throughout adulthood.

For men, four trajectories of labor participation were in the preferred estimates, as shown in Panel (b) in Fig. 1. The first trajectory is characterized by consistent participation in the labor market, even after age 60, consisting of 52.4 % of the sample. Next, we have a group (19.7 %) that had a high likelihood of being employed until age 65 (the legal retirement age for men), at which age the probability decreased perhaps due to retirement. The “exit from the labor-force around age 50″ path, followed by a 17.5 % of men in the sample, represents workers that were in the labor-force until around age 50 and left the labor-force after that age. Lastly, we have a “declining participation” group, with a low likelihood of being part of the labor-force throughout adulthood. This trajectory includes only 10.4 % of the men.

Table 2 shows descriptive characteristics of these groups. Women with consistent low participation in the labor market have worked, in average, less than a year and have the lowest average grades of educational attainment (7.7 grades). In addition, 44.7 % of women in this group report poor childhood socioeconomic status and 27.9 % report limitations in ADLs (Activities of Daily Living) (27.9 %).

In contrast, women that enter the labor-force around age 30 have the highest average number of years employed (30.0 years) and the highest average grades of education (9.3 grades). Women in this group are similar to those with consistent participation until retirement age, with 29.3 years of employment on average and 9.3 grades of educational attainment. Despite these similarities, there are notable differences in health: the latter group shows a lower percentage of women with ADL limitations (17.3 %) compared to the former (29.8 %). It is also interesting to note that women with late entrance to the labor-force have a higher proportion of never having been married.

In general, groups with higher employment participation tend to have more grades of schooling. Regarding childhood SES and physical limitations, the associations with employment are less clear.

The employment trajectories for men show a clearer gradient between health, as measured by limitations in activities of daily living (ADL), and labor-force participation. Men with poorer health are concentrated in the groups with lower and declining participation in the labor-force. The “declining-participation” group, for example, is characterized by low employment, with an average of 11.2 years of employment, and the lowest level of education (7.1 grades). This group also has a high percentage of individuals reporting poor childhood socioeconomic status (53.9 %) and a high prevalence of ADL limitations (15.6 %). Similarly, men who exit the labor-force around age 50 worked an average of 25.6 years and have slightly higher education (7.6 grades), but this group shows the highest rates of ADL limitations (31.6 %) and childhood socioeconomic disadvantages (42.0 %).

In contrast, men who work until retirement age were employed more years (30.0 on average) and are better educated (9.0 grades), and their health outcomes were notably better, with fewer ADL limitations (13.8 %). The “consistent high-participation” trajectory, which includes more than half the male sample (52.4 %), represents men with the highest number of years employed (34.7 years). This group also reports the lowest rates of ADL limitations (8.1 %), despite a sizable portion (56.9 %) coming from disadvantaged childhoods. Contrary to women, there is a higher proportion of men who have never been married in the trajectories characterized by less engagement in the labor-force.

Results on the GBTM for informal and full-time employment are available in Fig. A2 and A3 in the Appendix. Almost half of the women in the sample follow a trajectory of low informality during adulthood with an increase after retirement age. Men also tend to follow trajectories characterized by low informality. 39.5 % of women and 65.0 % of men follow full-time employment trajectories. Contrary to labor-force participation, results show no strong differences by gender in trajectories of informality and full-time employment.

### 3.3. Second stage: association between employment trajectories and cognitive function for older adults

**Fig. 2.**
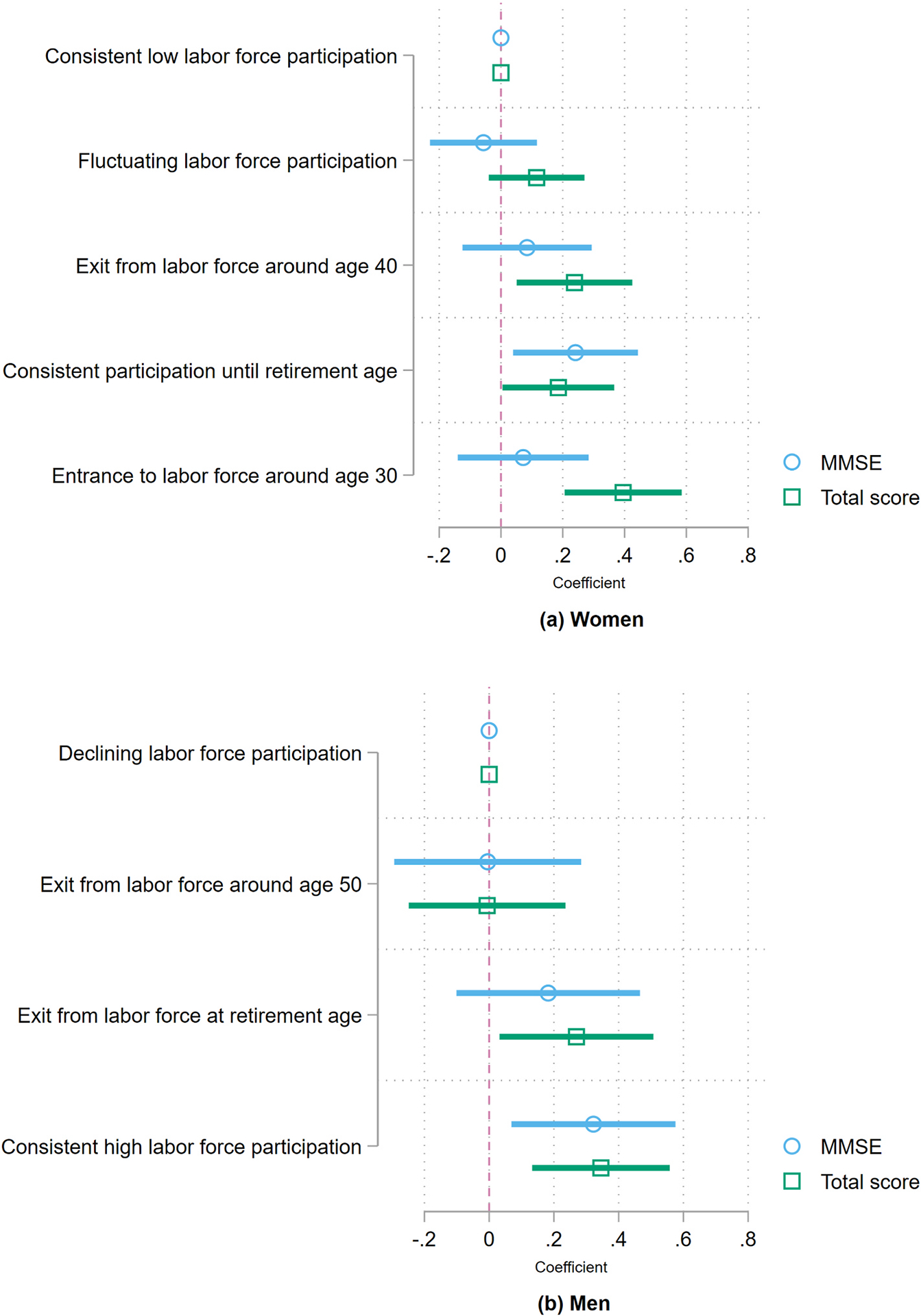
Association between labor-force participation and cognitive function for older adults Notes: (i) Coefficients come from estimates in Table A5 and Table A6 for women and men, respectively. (ii) All models incorporate survey weights. (iii) Models control for age, age squared, grades of educational attainment, grades of educational attainment squared, childhood socioeconomic conditions, lifetime marital status and physical limitations. (iv) Confidence interval at a 95 % confidence level.

With the identified employment trajectories for women and men, I estimate how they associate with cognitive function in later life. Fig. 2 shows the results from Equation (4), where the right-side variables are the trajectories estimated through GBTM and the dependent variables are MMSE or the total cognitive score. These models control for age, age squared, grades of educational attainment, grades of educational attainment squared, childhood socioeconomic conditions, lifetime marital status and limitations in Activities of Daily Living, and incorporate survey weights. Full tables are available in the Appendix.

Fig. 2, panel (a), shows that women that enter the labor-force after age 30 and continue to work have better total cognitive scores in 2019, compared to women with consistent low participation. Moreover, women that exit the labor-force at retirement age (60 years) have better MMSE and total cognitive scores, contrasted with women with lower engagement in the labor-force. Similar patterns can be seen for women who exit the labor-force at around age 40 and for women with more fluctuating trajectories, even though the latter is not statistically significant. To provide context for these associations, consider the following: entering the labor-force after age 30 and continuing to work after retirement age, which is characterized by more years of employment, is associated with 0.40 additional standard deviations in the total cognitive score compared to women with low labor-force participation.

Results for men are even stronger, as shown in Panel (b) in Fig. 2. Having a consistent high participation in the labor-force is associated with higher MMSE scores and total cognitive scores compared to having a declining participation trajectory. Moreover, men that exited the labor-force at retirement age also have significantly higher performance in the total cognitive measure compared to men whose likelihood of participating in the labor-force declines during adulthood. To put these numbers in perspective, consistent participation is associated with an additional 0.35 standard deviations in the total cognitive score compared to men with declining participation, as seen in Table A6.

**Fig. 3.**
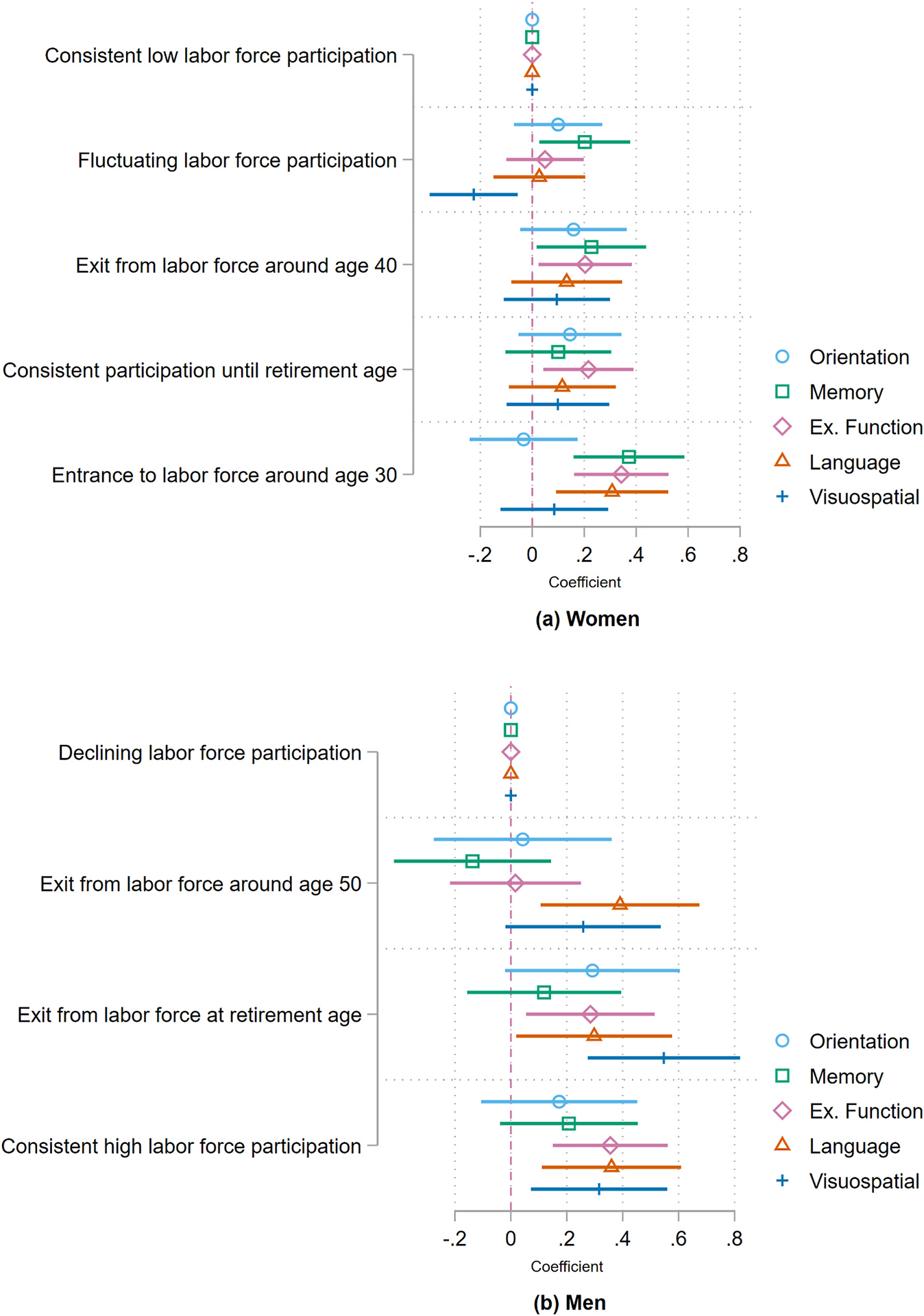
Association between labor-force participation and cognitive function for older adults by domain Notes: (i) Coefficients come from estimates in Table A5 and Table A6 for women and men, respectively. (ii) All models incorporate survey weights. (iii) Models control for age, age squared, grades of educational attainment, grades of educational attainment squared, childhood socioeconomic conditions, lifetime marital status and physical limitations. (iv) Confidence interval at a 95 % confidence level.

To explore how different cognitive domains relate to employment trajectories, I also estimate linear regression models for the five domains. Fig. 3, panel (a) presents the results for women. As can be seen, there are positive associations between trajectories characterized by longer employment and some specific cognitive domains. In particular, women who enter the labor-force after age 30 score better on memory and executive function, compared to women with low participation. Besides a few trajectories, language and visuospatial domains do not appear to be associated with employment histories. Panel (b) shows the association between employment trajectories and different cognitive domains for men. Results appear to be strong for men, especially for executive function, language and visuospatial.

### 3.4. Supplementary analysis

In supplementary analysis, I further examine whether informality and full-time employment trajectories are linked to cognitive performance in later life. For these analyses, I first use GBTM to estimate informal work and full-time employment histories. Results for the trajectories can be found in Appendix Figures A2 and A3. Next, I analyze the associations between these histories and cognitive scores, separately for men and women. The results, found in Tables A7 through A10 for the MMSE, the total score and the five specific cognitive domains in the Appendix, reveal no significant differences in cognitive scores by different informal or full-time employment trajectories.

## 4. Discussion

This study explores the associations between employment histories and later-life cognitive function for older adults in Chile. The findings highlight the importance of employment across the lifespan for predicting cognitive function in later life among older adults.

Descriptive statistics show clear patterns in which more education and fewer health limitations are associated with longer participation in the labor-force. Men who exit the labor-force younger or experience declining participation tend to have lower education, worse health, and more disadvantaged childhoods, while those working until retirement age generally fared better. In addition, descriptive statistics show that women who stay in the labor-force after the legal retirement age come from poorer childhood socioeconomic status than those who leave the labor-force around retirement age and have more physical limitations. These patterns suggest that continued employment may arise in part from financial necessity, particularly in cases with low contributions to individual pension accounts. Research in Chile supports this, showing that extended work after retirement age often involves self-employment, especially among those with more informal employment trajectories (Madero-Cabib and Biehl, 2021). Since informal employment is typically associated with no or minimal contributions to pension accounts, individuals following these trajectories may lack sufficient pension savings to fully retire, leading them to continue working to supplement their income.

A key finding is the consistent association between prolonged and stable employment and better cognitive outcomes at older ages, which persists even after adjusting for socioeconomic and health variables, suggesting that employment itself may play a protective role against cognitive decline. For example, results for women show that at the average grade of educational attainment (8.3 grades), an additional grade is associated with a 0.11 standard deviation increase in total cognition. This implies that the cognitive benefit of consistent labor-force participation, compared to little or no participation, is nearly equivalent to the benefit associated with 1.7 additional grades of education.

For both men and women, trajectories characterized by stable, long-term participation in the workforce, especially for those working up until retirement age or thereafter, are associated with significantly higher cognitive scores across multiple domains, including memory and executive function. These results suggest that work might stimulate different areas of the brain that help with cognitive performance in older age. These trajectories, indicative of consistent cognitive stimulation and social interaction, may contribute to cognitive reserve, a theory that posits such experiences can delay the onset of cognitive impairments (Stern, 2002).

Conversely, trajectories involving early exits from the workforce or sporadic employment histories are linked to poorer cognitive outcomes for older adults. Particularly for women, the low-participation trajectories correlate with lower cognitive scores. This may reflect not only reduced cognitive engagement but also potential economic and psychosocial stresses associated with earlier and less stable employment, which can adversely affect cognitive health (Braveman et al., 2011). It is important to mention that people whose employment trajectories were characterized by less engagement in the labor-force also have a higher proportion of physical limitations. This might have prevented them from working longer. However, even after controlling by ADL limitations, results still show strong associations between longer participation in the labor-force and cognition.

The analysis reveals gender differences in the impact of employment on different cognitive domains. Men benefit more distinctly from continuous employment, with stronger associations observed across several cognitive domains compared to women. These differences might be attributed to the nature of the jobs held, where men might have had roles involving complex problem-solving and decision-making that provide greater cognitive stimulation (Pekkarinen and Vartiainen, 2004). For women, those following trajectories with increased labor-force participation in midlife showed better cognitive outcomes. This finding could reflect that these women, who I hypothesize engaged in the workforce later due to childbearing responsibilities, benefited from the cognitive and social engagement provided by employment during a critical period of their adult lives.

These findings are consistent with existing literature, both in high-income countries (Andel et al., 2005; Kajitani et al., 2017; Kobayashi and Feldman, 2019; Pool et al., 2016) and middle-income settings (Yu et al., 2024). Moreover, additional evidence in Europe has found that repeated periods of unemployment are linked to worse cognition (Wahrendorf et al., 2019, 2021), which is consistent with these results, especially for men. Nevertheless, Leist et al. (2013) find that the reason for unemployment matters for cognition, which might explain why I still find that women with fluctuating labor-force trajectories have similar cognitive scores in memory compared to women who spend more time in the workforce.

Regarding informal work, I find no significant lower scores in those respondents who followed more vulnerable trajectories in the informal sector. These results are not consistent with the idea that financial security and access to healthcare are protectors of health in older ages, as suggested by prior research (Braveman et al., 2011). These findings might be explained by the expansion in labor and health policies, such as universal health care, which might have compensated for the lack of financial security provided by formal work.

Findings on part-time employment contrast with what has been found in Europe (Bertogg and Leist, 2023), as I do not find that part-time employment is associated with better cognition for women. In Chile, part-time employment might be an important option for less-educated women for whom childcare access is limited (Contreras et al., 2011). As a result, selection into part-time versus full-time employment could influence cognition as well, making it difficult to isolate the association between part-time employment and cognition.

The Chilean case provides an interesting context to study how employment trajectories are associated with cognition later in life. Older adults included in the analysis experienced an economic transition that moved Chile from a middle-to a high-income country. The implications of these processes for cognitive aging are not clear and more research is needed. However, these results might be useful for aging countries that experience similar economic progress. In addition, these findings highlight the importance of considering the long-term cognitive health implications of employment policies and practices, especially in the context of improving economic conditions. Encouraging policies that extend working life, perhaps through flexible working arrangements or phased retirement, could potentially enhance cognitive performance among older adults. Furthermore, interventions aimed at supporting women to enter or re-enter the workforce could not only improve economic outcomes but also potentially enhance their cognitive outcomes in later life. Such policies would be particularly relevant in contexts like Chile, where traditional gender roles have historically influenced women’s labor-force participation (Contreras and Plaza, 2010). Nevertheless, it is important to keep in mind that the type and quality of employment vary significantly, and not all jobs offer the same health benefits. Prolonged employment may be detrimental in some cases, such as for people in certain employment sectors (Arora et al., 2021; Devillanova et al., 2019), and people with precarious employment (Hajat et al., 2024). Therefore, a better understanding of the pathways through which paid work affects cognition in older ages is crucial.

### 4.1. Limitations

While this study offers insightful findings, it is not without limitations. The gap between employment histories and cognitive assessments may overlook the immediate effects of recent employment changes on cognitive function. Additionally, the nature of the data limits the ability to infer causality. Future research could benefit from longitudinal designs that track cognitive changes more closely relative to employment transitions to better understand the dynamics over time and permit exploring reverse causality in which cognition affects employment trajectories. Furthermore, due to data limitations, I am unable to distinguish between fluid and crystallized cognition, which may be differentially affected by employment patterns. This restricts the ability to test hypotheses about which cognitive processes are most responsive to labor-force participation experiences.

Lastly, it is essential to acknowledge the potential role of health selection bias. Since the analysis is based on a sample of respondents who are alive and participated in the cognitive assessments, there could be inherent selection bias towards individuals with better health and cognitive function. Consequently, the findings may not fully capture the experiences and outcomes of those with poorer health or cognitive impairment, limiting the generalizability of the results to the broader population.

## 5. Conclusion

This study contributes valuable evidence to the discussion on aging, work, and cognitive health, suggesting that not only does work matter, but length and continuity of work are crucial in predicting cognitive outcomes at older ages. This reinforces the need for policies that enhance job quality and stability as strategic measures to support cognitive health when population becomes older. Moreover, future research that explores the qualitative aspects of employment, such as occupation, job satisfaction and perceived job strain, could provide deeper insights into how the type and quality of work impact cognitive health beyond the mere categorical distinctions of employment status.

## Supplementary Material

1

## Figures and Tables

**Table 1:** Summary statistics by sex.

	Women		Men	
	(1)	(2)	(3)	(4)
	Mean	Obs.	Mean	Obs.
**Cognition**				
Mini-Mental State Examination (MMSE)	25.5	845	25.8	663
Total cognitive score	237.4	845	233.8	663
Orientation	10.3	845	10.3	663
Memory	96.6	845	90.2	663
Executive function	92.1	845	94.5	663
Language	29.4	845	29.5	663
Visuospatial	9.0	845	9.3	663
**Employment characteristics**				
N° of years employed	10.8	845	29.8	663
N° of years with informal employment	7.3	674	12.5	656
N° of years with a full-time job	12.0	674	25.9	656
**Other covariates**				
Age	67.7	845	67.4	663
Grades of educational attainment (%)	8.3	845	8.8	663
Poor childhood SES (%)	45.7	845	51.9	663
Never married (%)	11.8	845	10.5	663
One or more ADLs limitations (%)	25.3	845	14.4	663

Notes: (i) Descriptive statistics are weighted using survey weights.

**Table 2:** Summary statistics for labor-force participation trajectories.

	(1)	(2)	(3)	(4)	(5)	(6)
	%	N° of years employed	Grades of educational attainment	% poor childhood SES	% never married	% 1+ ADLs limitation
**Women (N = 845)**						
1. Consistent low participation in labor-force	49.0	0.5	7.7	44.7	7.1	27.9
2. Fluctuating participation in labor-force	17.0	10.3	8.6	43.5	5.7	24.2
3. Exit from labor-force around age 40	10.5	17.7	8.8	34.7	16.5	19.7
4. Consistent participation until retirement age	12.6	29.3	9.3	51.2	22.5	17.3
5. Entrance to labor-force around age 30	10.9	30.0	9.3	57.4	25.4	29.8
**Men (N = 663)**						
1. Declining participation in labor-force	10.4	11.2	7.1	53.9	25.4	15.6
2. Exit from labor-force around age 50	17.5	25.6	7.6	42.0	8.8	33.2
3. Exit from labor-force at retirement age	19.7	30.0	9.0	46.4	4.7	13.8
4. Consistent high participation in labor-force	52.4	34.7	9.5	56.9	10.3	8.1

Notes: (i) Trajectories come from Group-Based Trajectory Model. (ii) Model selection was based on BIC. (iii) Descriptive statistics are weighted using survey weights.

## Data Availability

The data used in this study are publicly available. The Social Protection Survey data can be accessed through the Undersecretary of Social Security at https://www.previsionsocial.gob.cl. The Chile-Cog data, including the imputed cognitive scores, are available through the Gateway to Global Aging Data at https://g2aging.org.

## Appendix A. Supplementary data

Supplementary data to this article can be found online at https://doi.org/10.1016/j.socscimed.2025.118281.
