# Supplementary material for: The predictive power of employment trajectories on cognition of older adults: Evidence from Chile
Source: Soc Sci Med. Author manuscript; Available in PMC 2026 Apr 7. (PMC13056228; doi:10.1016/j.socscimed.2025.118281)
Supplement: 1 [file NIHMS2162033-supplement-1.pdf]

Appendix

Tables

Table A1
Descriptive statistics - Women

	Obs.	Mean/%	Std. Dev.	Min.	Max.
**Cognition**					
Mini-Mental State Examination (MMSE)	845	25.5	2.8	11	30
Total cognitive score	845	237.4	61.0	56	391
Orientation	845	10.3	1.0	3	11
Memory	845	96.6	27.5	17	182
Executive function	845	92.1	34.7	6	176
Language	845	29.4	5.5	10	50
Visuospatial	845	9.0	2.3	1	12
**Employment characteristics**					
N^o of years employed	845	10.8	12.4	0	36
N^o of years with informal employment	674	7.3	9.2	0	36
N^o of years with a full-time job	674	12.0	11.4	0	36
**Other covariates**					
Age	845	67.7	5.5	60	79
Grades of educational attainment	845	8.3	3.8	0	23
Poor childhood SES	397	45.7			
Never married	133	11.8			
One or more ADL limitation	182	25.3			

Notes: (i) The following variables were created with information collected up until 2016: number of years employed, number with an informal employment and number of years with a full-time job. (ii) Limitations in the Activities of Daily Living (ADL) comes from the 2015/2016 wave. (iii) Descriptive statistics are weighted using survey weights.

Table A2

Descriptive statistics - Men

	Obs.	Mean/%	Std. Dev.	Min.	Max.
**Cognition**					
Mini-Mental State Examination (MMSE)	663	25.8	3.1	14	30
Total cognitive score	663	233.8	63.9	55	382
Orientation	663	10.3	1.1	3	11
Memory	663	90.2	28.4	22	172
Executive function	663	94.5	36.6	2	181
Language	663	29.5	5.9	11	48
Visuospatial	663	9.3	2.5	1	12
**Employment characteristics**					
N^o of years employed	663	29.8	8.2	0	36
N^o of years with informal employment	656	12.5	12.7	0	36
N^o of years with a full-time job	656	25.9	10.4	0	36
**Other covariates**					
Age	663	67.4	5.6	60	79
Grades of educational attainment	663	8.8	4.0	0	17
Poor childhood SES	355	51.9			
Never married	53	10.5			
One or more ADL limitation	101	14.4			

Notes: (i) The following variables were created with information collected up until 2016: number of years employed, number with an informal employment and number of years with a full-time job. (ii) Limitations in the Activities of Daily Living (ADL) comes from the 2015/2016 wave. (iii) Descriptive statistics are weighted using survey weights.

Table A3

Model fit for different specifications

	Age polynomial	N° of groups	BIC
Women	Quadratic	2	27,001.0
	Quadratic	3	24,303.4
	Quadratic	4	22,202.6
	Quadratic	5	20,980.6
	Cubic	2	26,859.1
	Cubic	3	24,177.6
	Cubic	4	22,030.3
	Cubic	5	20,805.4
Men	Quadratic	2	13,623.5
	Quadratic	3	12,327.4
	Quadratic	4	11,659.3
	Quadratic	5	11,695.1
	Cubic	2	13,632.7
	Cubic	3	12,341.6
	Cubic	4	11,585.2
	Cubic	5	11,693.1

Table A4

Group membership probabilities for selected models

	%	Mean posterior probabilities	Entropy
**Women (N=845)**			
1. Consistent low participation in labor force	49.0	0.988	
2. Fluctuating participation in labor force	17.0	0.981	
3. Exit from labor force around age 40	12.6	0.977	0.969
4. Consistent participation until retirement age	12.6	0.979	
5. Entrance to labor force around age 30	10.9	0.964	
**Men (N=663)**			
1. Declining participation in labor force	10.4	0.980	
2. Exit from labor force around age 50	17.5	0.971	0.830
3. Exit from labor force at retirement age	19.7	0.957	
4. Consistent high participation in labor force	52.4	0.897	

Notes: (i) Distributions are weighted using survey weights.

Table A5

Associations between labor force participation and cognitive function for women

	(1) MMSE	(2) Total score	(3) Orientation	(4) Memory	(5) Executive function	(6) Language	(7) Visuospatial
**Group-based employment trajectory (ref: Consistent labor force participation)**							
Fluctuating labor force participation	-0.06	0.12	0.10	0.20**	0.05	0.03	-0.23***
	(0.09)	(0.08)	(0.09)	(0.09)	(0.08)	(0.09)	(0.09)
Exit from labor force around age 40	0.08	0.24**	0.16	0.23**	0.20**	0.13	0.09
	(0.11)	(0.10)	(0.10)	(0.11)	(0.09)	(0.11)	(0.10)
Consistent participation until retirement age	0.24**	0.19**	0.15	0.10	0.22**	0.12	0.10
	(0.10)	(0.09)	(0.10)	(0.10)	(0.09)	(0.10)	(0.10)
Entrance to labor force around age 30	0.07	0.40***	-0.03	0.37***	0.34***	0.31***	0.08
	(0.11)	(0.10)	(0.11)	(0.11)	(0.09)	(0.11)	(0.11)
**Covariates**							
Age (mean-centered)	-0.02***	-0.04***	-0.01**	-0.02***	-0.04***	-0.02**	-0.01**
	(0.01)	(0.01)	(0.01)	(0.01)	(0.01)	(0.01)	(0.01)
Age (mean-centered, squared)	0.00	-0.00	0.00	0.00	-0.00	-0.00**	-0.00
	(0.00)	(0.00)	(0.00)	(0.00)	(0.00)	(0.00)	(0.00)
Grades of educational attainment	0.08**	0.06**	0.09***	0.03	0.08***	0.02	0.05*
	(0.03)	(0.03)	(0.03)	(0.03)	(0.03)	(0.03)	(0.03)
Grades of educational attainment (squared)	-0.00	0.00*	-0.00	0.00	0.00	0.00	0.00
	(0.00)	(0.00)	(0.00)	(0.00)	(0.00)	(0.00)	(0.00)
Poor childhood SES	-0.14**	-0.14**	0.03	-0.07	-0.17***	0.01	-0.11*
	(0.07)	(0.06)	(0.06)	(0.07)	(0.06)	(0.07)	(0.06)
One or more ADL limitation	-0.04	-0.20***	-0.14*	-0.15**	-0.18***	-0.18**	-0.18**
	(0.07)	(0.07)	(0.07)	(0.07)	(0.06)	(0.07)	(0.07)
Never married	-0.12	-0.04	-0.06	0.00	-0.07	-0.05	-0.03
	(0.10)	(0.09)	(0.10)	(0.10)	(0.09)	(0.10)	(0.10)
Constant	-0.67***	-0.70***	-0.52***	-0.39***	-0.81***	-0.29*	-0.63***
	(0.15)	(0.13)	(0.14)	(0.15)	(0.13)	(0.15)	(0.14)
Observations	845	845	845	845	845	845	845

Notes: (i) Reference categories are good childhood SES, no ADL limitations and ever married. (ii) All models incorporate survey weights. (iii) Standard errors on parenthesis. ***, ** and * indicate statistical significance at the 99%, 95% and 90%, respectively.

Table A6
Associations between labor force participation and cognitive function for men

	(1) MMSE	(2) Total score	(3) Orientation	(4) Memory	(5) Executive function	(6) Language	(7) Visuospatial
**Group-based employment trajectory (ref: Declining labor force participation)**							
Exit from labor force around age 50	-0.00	-0.01	0.04	-0.14	0.02	0.39***	0.26*
	(0.15)	(0.12)	(0.16)	(0.14)	(0.12)	(0.14)	(0.14)
Exit from labor force at retirement age	0.18	0.27**	0.29*	0.12	0.28**	0.30**	0.55***
	(0.14)	(0.12)	(0.16)	(0.14)	(0.12)	(0.14)	(0.14)
Consistent high labor force participation	0.32**	0.35***	0.17	0.21*	0.36***	0.36***	0.32**
	(0.13)	(0.11)	(0.14)	(0.13)	(0.10)	(0.13)	(0.12)
**Covariates**							
Age (mean-centered)	-0.03***	-0.05***	-0.03***	-0.04***	-0.05***	-0.04***	-0.03***
	(0.01)	(0.01)	(0.01)	(0.01)	(0.01)	(0.01)	(0.01)
Age (mean-centered, squared)	0.00	0.00	-0.00	0.00	0.00	0.00	-0.00
	(0.00)	(0.00)	(0.00)	(0.00)	(0.00)	(0.00)	(0.00)
Grades of educational attainment	0.12***	0.08***	0.04	0.02	0.11***	0.04	0.22***
	(0.04)	(0.03)	(0.04)	(0.04)	(0.03)	(0.04)	(0.04)
Grades of educational attainment (squared)	-0.00	0.00	0.00	0.00*	0.00	0.00	-0.01***
	(0.00)	(0.00)	(0.00)	(0.00)	(0.00)	(0.00)	(0.00)
Poor childhood SES	0.06	-0.09	0.07	0.02	-0.14**	-0.14*	-0.19**
	(0.08)	(0.07)	(0.09)	(0.08)	(0.06)	(0.08)	(0.08)
One or more ADL limitation	-0.03	-0.12	-0.22*	-0.12	-0.13	0.10	-0.05
	(0.11)	(0.09)	(0.12)	(0.11)	(0.09)	(0.11)	(0.10)
Never married	-0.34***	-0.52***	-0.06	-0.32***	-0.59***	-0.26**	-0.39***
	(0.12)	(0.10)	(0.13)	(0.12)	(0.10)	(0.12)	(0.12)
Constant	-1.10***	-1.11***	-0.47**	-0.70***	-1.15***	-0.80***	-1.38***
	(0.20)	(0.17)	(0.22)	(0.20)	(0.17)	(0.20)	(0.20)
Observations	663	663	663	663	663	663	663

Notes: (i) Reference categories are good childhood SES, no ADL limitations and ever married. (ii) All models incorporate survey weights. (iii) Standard errors on parenthesis. ***, ** and * indicate statistical significance at the 99%, 95% and 90%, respectively.

Table A7

Associations between informal employment trajectories and cognitive function for women

	(1) MMSE	(2) Total score	(3) Orientation	(4) Memory	(5) Executive function	(6) Language	(7) Visuospatial
**Group-based informality trajectory (ref: Low informality path and increase at retirement age)**							
2. Midlife increase in informality	0.01	-0.01	-0.03	0.11	-0.08	0.05	-0.31***
	(0.09)	(0.09)	(0.09)	(0.10)	(0.08)	(0.10)	(0.09)
3. Midlife decrease in informality	0.02	-0.03	-0.00	0.02	-0.06	0.03	-0.00
	(0.11)	(0.11)	(0.11)	(0.12)	(0.10)	(0.12)	(0.11)
4. High informality path until retirement age	0.09	0.14	-0.02	0.07	0.14*	0.26***	0.04
	(0.09)	(0.09)	(0.09)	(0.10)	(0.08)	(0.09)	(0.09)
**Covariates**							
Age (mean-centered)	-0.01*	-0.04***	-0.01**	-0.02***	-0.04***	-0.02***	-0.00
	(0.01)	(0.01)	(0.01)	(0.01)	(0.01)	(0.01)	(0.01)
Age (mean-centered, squared)	0.00	-0.00	0.00	0.00	-0.00*	-0.00	0.00
	(0.00)	(0.00)	(0.00)	(0.00)	(0.00)	(0.00)	(0.00)
Grades of educational attainment	0.12***	0.07**	0.14***	0.04	0.08***	0.04	0.08**
	(0.03)	(0.03)	(0.03)	(0.03)	(0.03)	(0.03)	(0.03)
Grades of educational attainment (squared)	-0.00	0.00*	-0.01***	0.00	0.00*	0.00	0.00
	(0.00)	(0.00)	(0.00)	(0.00)	(0.00)	(0.00)	(0.00)
Poor childhood SES	-0.12	0.04	-0.01	0.06	-0.02	0.22***	0.09
	(0.07)	(0.07)	(0.07)	(0.08)	(0.06)	(0.08)	(0.07)
One or more ADL limitation	-0.11	-0.23***	-0.22***	-0.16*	-0.20***	-0.25***	-0.25***
	(0.08)	(0.08)	(0.08)	(0.09)	(0.07)	(0.08)	(0.08)
Never married	-0.02	0.04	0.00	0.05	0.02	0.06	-0.04
	(0.10)	(0.09)	(0.09)	(0.10)	(0.09)	(0.10)	(0.10)
Constant	-0.81***	-0.73***	-0.61***	-0.47***	-0.75***	-0.56***	-0.77***
	(0.16)	(0.15)	(0.15)	(0.17)	(0.14)	(0.16)	(0.16)
Observations	674	674	674	674	674	674	674

Notes: (i) Reference categories are good childhood SES, no ADL limitations and ever married. (ii) All models incorporate survey weights. (iii) Standard errors on parenthesis. ***, ** and * indicate statistical significance at the 99%, 95% and 90%, respectively.

Table A8

Associations between informal employment trajectories and cognitive function for men

	(1) MMSE	(2) Total score	(3) Orientation	(4) Memory	(5) Executive function	(6) Language	(7) Visuospatial
**Group-based informality trajectory (ref: Low informality path)**							
2. Low informality path and increase at age 50	0.12	0.02	-0.04	-0.07	0.06	0.16	0.09
	(0.10)	(0.09)	(0.11)	(0.10)	(0.08)	(0.10)	(0.10)
3. Decrease at age 30 and increase at retirement age	0.11	0.14	-0.03	0.30**	-0.02	0.10	0.06
	(0.13)	(0.11)	(0.14)	(0.12)	(0.10)	(0.13)	(0.12)
4. High informality path until retirement age	0.08	-0.01	-0.03	0.02	-0.06	0.14	-0.07
	(0.09)	(0.08)	(0.10)	(0.09)	(0.07)	(0.09)	(0.09)
**Covariates**							
Age (mean-centered)	-0.03***	-0.05***	-0.03***	-0.04***	-0.05***	-0.05***	-0.02***
	(0.01)	(0.01)	(0.01)	(0.01)	(0.01)	(0.01)	(0.01)
Age (mean-centered, squared)	-0.00	-0.00	-0.00*	-0.00	0.00	-0.00	-0.00
	(0.00)	(0.00)	(0.00)	(0.00)	(0.00)	(0.00)	(0.00)
Grades of educational attainment	0.14***	0.10***	0.05	0.03	0.13***	0.05	0.23***
	(0.04)	(0.03)	(0.04)	(0.04)	(0.03)	(0.04)	(0.04)
Grades of educational attainment (squared)	-0.00	0.00	-0.00	0.00	0.00	0.00	-0.01***
	(0.00)	(0.00)	(0.00)	(0.00)	(0.00)	(0.00)	(0.00)
Poor childhood SES	0.07	-0.11*	0.06	-0.02	-0.14**	-0.15*	-0.22***
	(0.08)	(0.07)	(0.09)	(0.08)	(0.06)	(0.08)	(0.08)
One or more ADL limitation	-0.12	-0.21**	-0.27**	-0.21**	-0.21**	0.10	-0.05
	(0.11)	(0.09)	(0.12)	(0.10)	(0.09)	(0.11)	(0.10)
Never married	-0.24*	-0.39***	0.05	-0.16	-0.48***	-0.24*	-0.46***
	(0.13)	(0.10)	(0.14)	(0.12)	(0.10)	(0.12)	(0.12)
Constant	-1.01***	-0.94***	-0.34*	-0.64***	-0.96***	-0.60***	-1.11***
	(0.19)	(0.16)	(0.20)	(0.18)	(0.15)	(0.19)	(0.18)
Observations	656	656	656	656	656	656	656

Notes: (i) Reference categories are good childhood SES, no ADL limitations and ever married. (ii) All models incorporate survey weights. (iii) Standard errors on parenthesis. ***, ** and * indicate statistical significance at the 99%, 95% and 90%, respectively.

Table A9

Associations between full-time employment trajectories and cognitive function for women

	(1) MMSE	(2) Total score	(3) Orientation	(4) Memory	(5) Executive function	(6) Language	(7) Visuospatial
**Group-based full-time trajectory (ref: part-time employment path)**							
2. Midlife hours decrease	-0.01	0.06	0.10	-0.02	0.10	0.16	-0.15
	(0.11)	(0.11)	(0.11)	(0.12)	(0.10)	(0.12)	(0.11)
3. Midlife hours increase	-0.08	0.14	0.16	0.08	0.13	0.33***	-0.14
	(0.12)	(0.11)	(0.12)	(0.13)	(0.11)	(0.12)	(0.12)
4. Full-time employment until retirement age	-0.02	0.06	-0.09	0.01	0.09	0.21*	-0.17
	(0.11)	(0.10)	(0.10)	(0.11)	(0.10)	(0.11)	(0.11)
**Covariates**							
Age (mean-centered)	-0.01*	-0.04***	-0.01*	-0.02***	-0.04***	-0.02***	-0.00
	(0.01)	(0.01)	(0.01)	(0.01)	(0.01)	(0.01)	(0.01)
Age (mean-centered, squared)	0.00	-0.00	0.00	0.00	-0.00*	-0.00	0.00
	(0.00)	(0.00)	(0.00)	(0.00)	(0.00)	(0.00)	(0.00)
Grades of educational attainment	0.12***	0.07**	0.13***	0.04	0.07**	0.04	0.06*
	(0.03)	(0.03)	(0.03)	(0.03)	(0.03)	(0.03)	(0.03)
Grades of educational attainment (squared)	-0.00	0.00*	-0.00***	0.00	0.00**	0.00	0.00
	(0.00)	(0.00)	(0.00)	(0.00)	(0.00)	(0.00)	(0.00)
Poor childhood SES	-0.11	0.05	-0.01	0.05	-0.00	0.23***	0.11
	(0.07)	(0.07)	(0.07)	(0.08)	(0.06)	(0.08)	(0.07)
One or more ADL limitation	-0.12	-0.22***	-0.24***	-0.16*	-0.20***	-0.23***	-0.29***
	(0.08)	(0.08)	(0.08)	(0.09)	(0.07)	(0.09)	(0.08)
Never married	-0.03	0.03	0.02	0.05	0.01	0.05	-0.03
	(0.10)	(0.09)	(0.09)	(0.10)	(0.09)	(0.10)	(0.10)
Constant	-0.75***	-0.75***	-0.63***	-0.42**	-0.81***	-0.64***	-0.65***
	(0.18)	(0.17)	(0.17)	(0.19)	(0.16)	(0.19)	(0.18)
Observations	674	674	674	674	674	674	674

Notes: (i) Reference categories are good childhood SES, no ADL limitations and ever married. (ii) All models incorporate survey weights. (iii) Standard errors on parenthesis. ***, ** and * indicate statistical significance at the 99%, 95% and 90%, respectively.

Table A10

Associations between full-time employment trajectories and cognitive function for men

	(1) MMSE	(2) Total score	(3) Orientation	(4) Memory	(5) Executive function	(6) Language	(7) Visuospatial
**Group-based full-time trajectory (ref: part-time employment until retirement age)**							
2. Midlife reduction of hours	0.10	-0.06	0.13	0.06	-0.16	-0.06	0.14
	(0.13)	(0.11)	(0.14)	(0.13)	(0.11)	(0.13)	(0.13)
3. Full-time employment path	0.28**	0.04	0.45***	0.11	-0.05	0.01	0.18
	(0.11)	(0.10)	(0.12)	(0.11)	(0.09)	(0.11)	(0.11)
**Covariates**							
Age (mean-centered)	-0.03***	-0.05***	-0.03***	-0.03***	-0.05***	-0.05***	-0.02***
	(0.01)	(0.01)	(0.01)	(0.01)	(0.01)	(0.01)	(0.01)
Age (mean-centered, squared)	-0.00	-0.00	-0.00*	-0.00	0.00	0.00	-0.00
	(0.00)	(0.00)	(0.00)	(0.00)	(0.00)	(0.00)	(0.00)
Grades of educational attainment	0.12***	0.10***	0.02	0.03	0.13***	0.05	0.22***
	(0.04)	(0.03)	(0.04)	(0.04)	(0.03)	(0.04)	(0.04)
Grades of educational attainment (squared)	-0.00	0.00	0.00	0.00*	0.00	0.00	-0.01***
	(0.00)	(0.00)	(0.00)	(0.00)	(0.00)	(0.00)	(0.00)
Poor childhood SES	0.08	-0.09	0.06	-0.00	-0.13**	-0.14*	-0.21***
	(0.08)	(0.07)	(0.09)	(0.08)	(0.06)	(0.08)	(0.08)
One or more ADL limitation	-0.12	-0.21**	-0.28**	-0.22**	-0.20**	0.11	-0.06
	(0.11)	(0.09)	(0.12)	(0.10)	(0.09)	(0.11)	(0.10)
Never married	-0.23*	-0.38***	0.07	-0.16	-0.47***	-0.21*	-0.49***
	(0.13)	(0.11)	(0.14)	(0.12)	(0.10)	(0.13)	(0.12)
Constant	-1.09***	-0.93***	-0.58***	-0.68***	-0.91***	-0.50**	-1.24***
	(0.20)	(0.16)	(0.21)	(0.19)	(0.16)	(0.19)	(0.19)
Observations	656	656	656	656	656	656	656

Notes: (i) Reference categories are good childhood SES, no ADL limitations and ever married. (ii) All models incorporate survey weights. (iii) Standard errors on parenthesis. ***, ** and * indicate statistical significance at the 99%, 95% and 90%, respectively.

Figures

Figure A1
Flowchart of sample selection

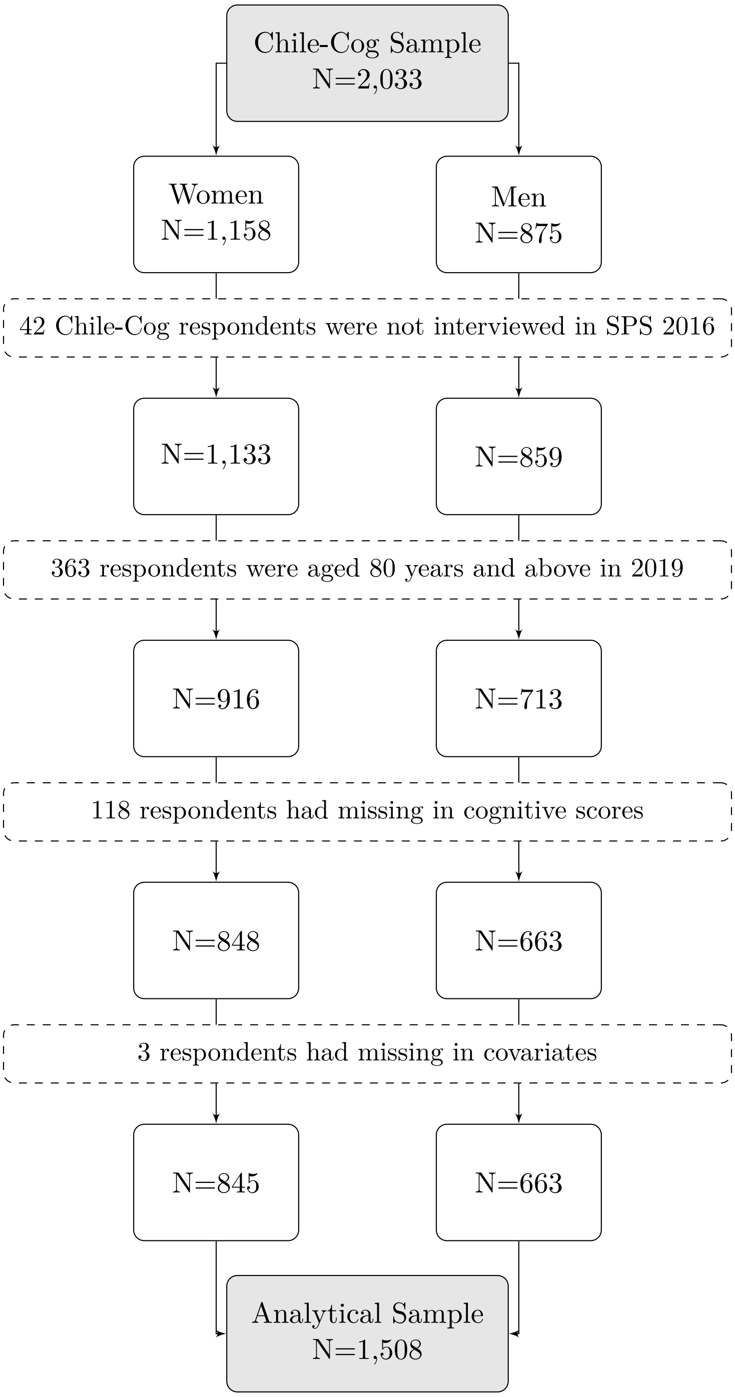

Figure A2
Informal employment trajectories

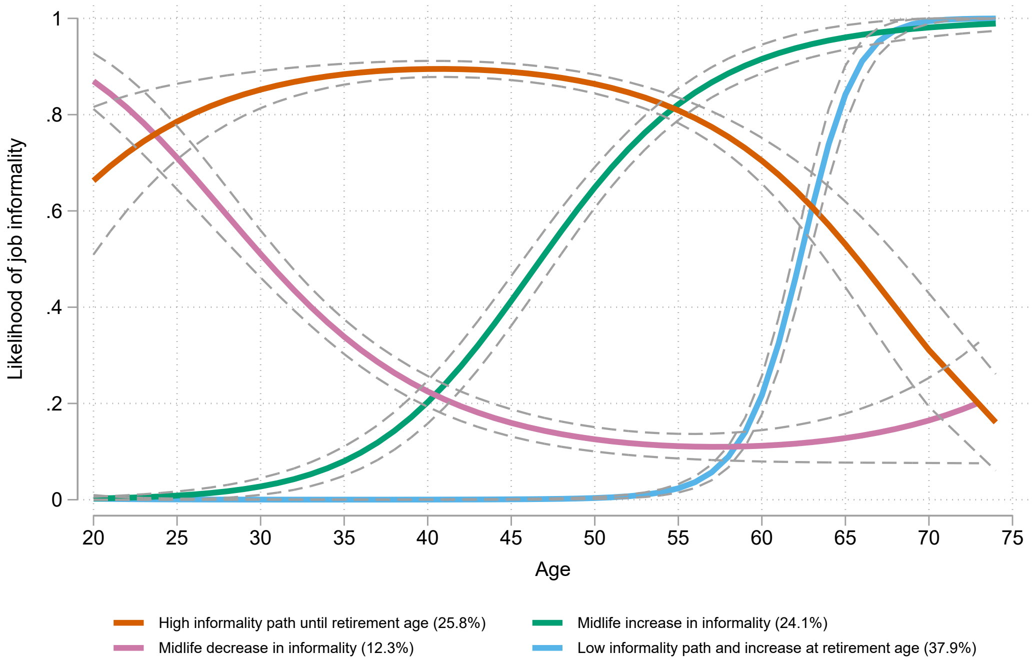

(a) **Women**

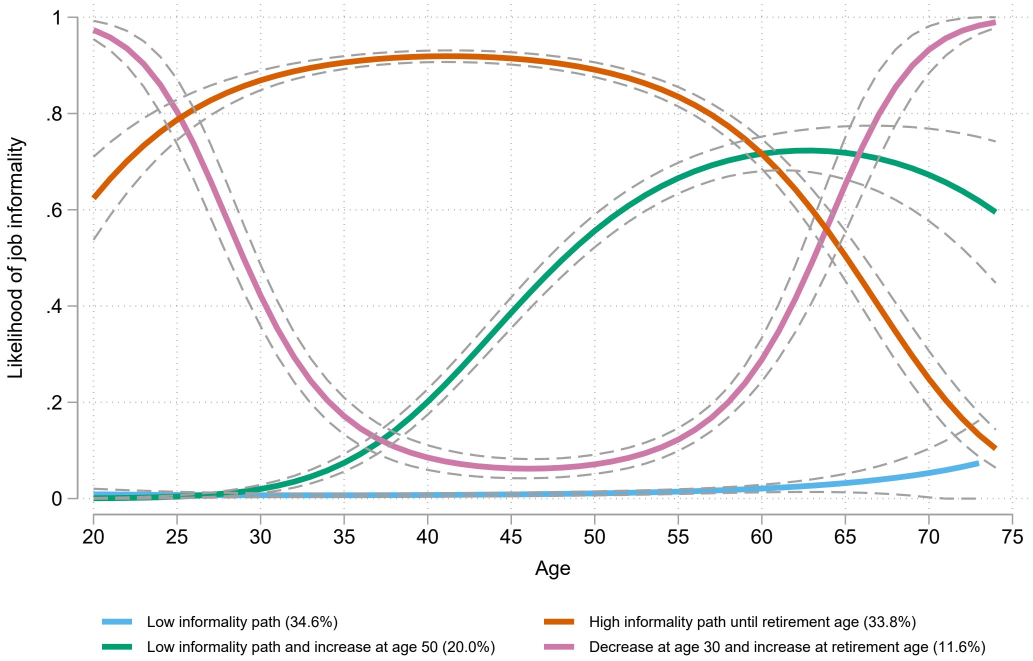

(b) **Men**

Notes: (i) Distributions are weighted using survey weights.

Figure A3

Full-time employment trajectories

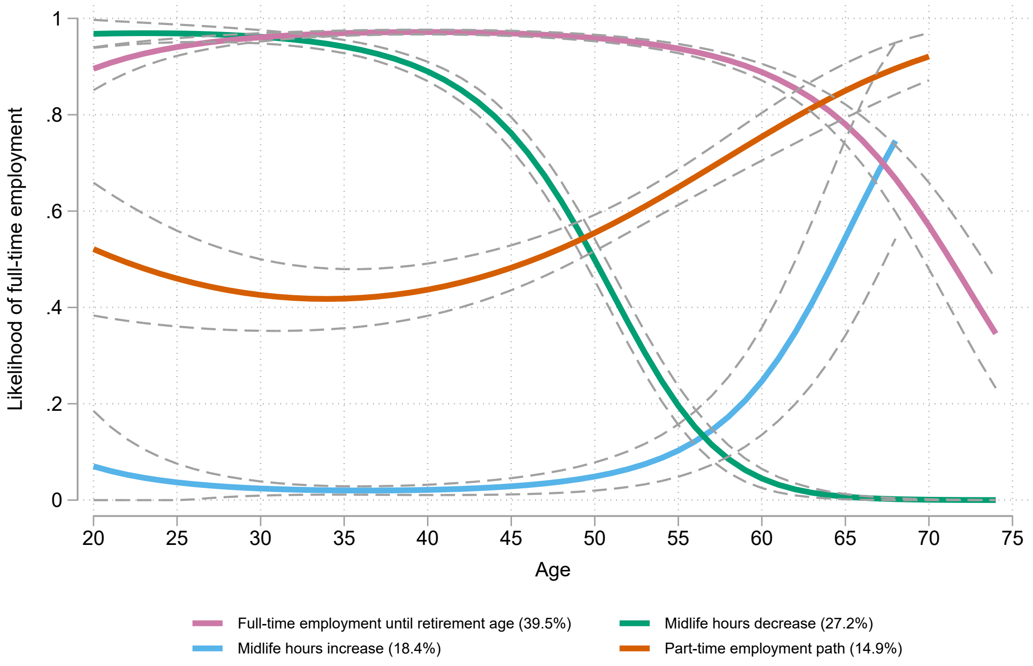

(a) **Women**

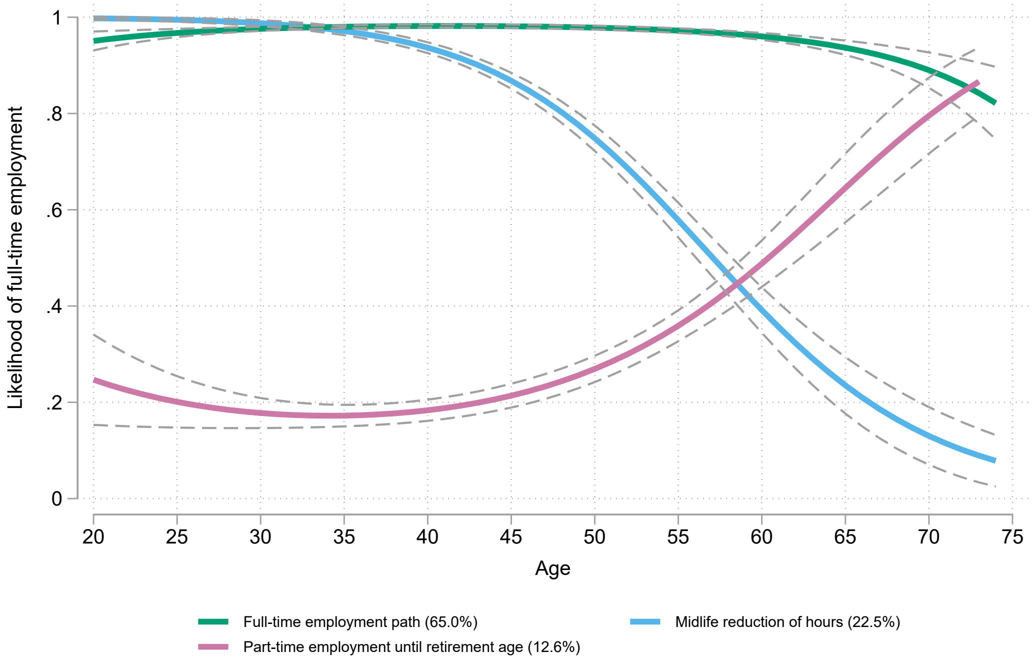

(b) **Men**

Notes: (i) Distributions are weighted using survey weights.
